# Gut Microbiota Cannot Compensate the Impact of (quasi) Aposymbiosis in *Blattella germanica*

**DOI:** 10.3390/biology10101013

**Published:** 2021-10-09

**Authors:** Maria Muñoz-Benavent, Amparo Latorre, Ester Alemany-Cosme, Jesús Marín-Miret, Rebeca Domínguez-Santos, Francisco J. Silva, Rosario Gil, Carlos García-Ferris

**Affiliations:** 1 Institute for Integrative Systems Biology, University of Valencia and Spanish Research Council, Paterna, 46980 Valencia, Spain; maria.munoz-benavent@uv.es (M.M.-B.); amparo.latorre@uv.es (A.L.); esacos@alumni.uv.es (E.A.-C.); jesus.marin@uv.es (J.M.-M.); rebeca.dominguez@uv.es (R.D.-S.); francisco.silva@uv.es (F.J.S.); 2 Genomic and Health Area, Foundation for the Promotion of Sanitary and Biomedical Research of the Valencia Region, 46020 Valencia, Spain; 3 Department of Biochemistry and Molecular Biology, University of Valencia, Burjassot, 46100 Valencia, Spain

**Keywords:** *Blattella germanica*, symbiosis, *Blattabacterium*, gut microbiota, rifampicin, aposymbiont

## Abstract

**Simple Summary:**

The German cockroach *Blattella germanica* is a good model to study complex symbiotic relationships because the following two symbiotic systems coexist in a single individual: the endosymbiont *Blattabacterium* (living inside specialized cells called bacteriocytes) and the gut microbiota. Although the role of the endosymbiont has been fully elucidated, the function of the gut microbiota remains unclear. The study of the gut microbiota will benefit from the availability of insects deprived of *Blattabacterium.* Our goal is to determine the effect of the removal (or, at least, the reduction) of the endosymbiont population on the cockroach’s fitness, in a normal gut microbiota community. For this purpose, we treated our cockroach population with rifampicin to decrease the amount of endosymbiont in the following generation. As the treatment also affects rifampicin-sensitive gut bacteria, we allowed it to recover for at least 20 days before sampling. We found that after this antibiotic treatment, the endosymbiont population remained extremely reduced and only the microbiota were able to recover, although it could not compensate for the endosymbiont role, and the host’s fitness was drastically affected. This accomplished reduction, however, is not homogenous and requires further study to develop stable quasi-aposymbiotic cockroaches.

**Abstract:**

*Blattella germanica* presents a very complex symbiotic system, involving the following two kinds of symbionts: the endosymbiont *Blattabacterium* and the gut microbiota. Although the role of the endosymbiont has been fully elucidated, the function of the gut microbiota remains unclear. The study of the gut microbiota will benefit from the availability of insects deprived of *Blattabacterium.* Our goal is to determine the effect of the removal (or, at least, the reduction) of the endosymbiont population on the cockroach’s fitness, in a normal gut microbiota community. For this purpose, we treated our cockroach population, over several generations, with rifampicin, an antibiotic that only affects the endosymbiont during its extracellular phase, and decreases its amount in the following generation. As rifampicin also affects gut bacteria that are sensitive to this antibiotic, the treatment was performed during the first 12 days of the adult stage, which is the period when the endosymbiont infects the oocytes and lacks bacteriocyte protection. We found that after this antibiotic treatment, the endosymbiont population remained extremely reduced and only the microbiota was able to recover, although it could not compensate for the endosymbiont role, and the host’s fitness was drastically affected. This accomplished reduction, however, is not homogenous and requires further study to develop stable quasi-aposymbiotic cockroaches.

## 1. Introduction

The cockroach *Blattella germanica* (Blattodea) is a good model to study complex symbiotic relationships because the following two symbiotic systems coexist in a single individual: the obligate endosymbiont *Blattabacterium*, a flavobacterium responsible for the synthesis of essential amino acids and the recycling of the host nitrogen reservoir [1], and very rich gut microbiota. Termites also belong to the order Blattodea. *Blattabacterium* is present in all cockroaches and the basal termite *Mastotermes darwiniensis*, indicating that the infection with the ancestral flavobacterium that gave rise to the endosymbiont occurred in the ancestor of termites and cockroaches. However, throughout evolution, and associated with a change to feeding on wood, the endosymbiont was lost in the rest of the termite lineages, being replaced by a specialized microbiota that, in addition to the endosymbiont functions, participated in lignocellulose degradation [2]. On the other hand, studies performed on insects that rely on obligate mutualistic endosymbionts to complement their nutrient-poor diets indicate that their microbial symbionts are composed of a few primary (obligate) and secondary (facultative) symbionts, mostly located in the insect body cavities, inside specialized cells called bacteriocytes, inside the gut epithelial cells or crypts (sac-like structures), but lack complex extracellular gut microbiota [3]. Thus, the reason why *Blattella* needs intestinal microbiota in addition to *Blattabacterium* remains unknown. Both symbiotic systems are located in different host compartments, the fat body and the hindgut, which impedes their direct crosstalk. Therefore, their communication or metabolic complementation, if existent, must be performed through the host.

The first 16S rRNA gene study on the bacterial gut microbiota of *B. germanica* through development [4] demonstrated that only *Blattabacterium* is present in the ootheca, confirming the absence of vertical transmission of the gut microbiota. The new-born nymphs are germ-free, and the bacterial load increases from nymphal instar one to two, and stays constant until the adult stage.

Later on, we tested the effect of different antibiotics (rifampicin [5], vancomycin and ampicillin [6], and kanamycin [7]) on the gut microbiota. In all four cases, a strong effect was observed on the treated generation, in terms of composition, diversity, and network complexity; however, only rifampicin reduced the *Blattabacterium* population [5], and its effect on the endosymbiont was observed in the following generation. This delayed action is explained by the biology of the endosymbiont, as it appears extracellularly in the ovaries at the adult stage until the infection of the mature oocytes, when the adult is 7–10 days old [8].

Aposymbiotic organisms, deprived of their endosymbiont, but in which the gut microbiota are not affected, would be useful to study the role of the gut microbiota and if they can complement (or substitute) *Blattabacterium*. Several approaches to produce aposymbiotic cockroaches (high temperature, or treatment with lysozyme and several antibiotics, which could be injected into the haemolymph or added in the food) were already tested in the middle of the 20th century, by the pioneers of the symbiosis studies [9,10]. These early works already recognized, by means of biochemical, physiological and microscopic techniques, that the presence of the endosymbiont is critical to the host’s fitness and reproduction. For this reason, we focused this study on obtaining quasi-aposymbiotic cockroaches (i.e., with the lowest possible endosymbiont load) using an antibiotic treatment, without disturbing the gut microbiota. We analysed the effects of the endosymbiont reduction on the other two symbiotic partners (host and gut microbiota), by analysing changes in the host’s fitness (developmental time, reproductive capability, and viability) and the gut microbiota composition.

## 2. Materials and Methods

### 2.1. Blattella germanica Rearing Conditions

A population of *B. germanica* originating from a laboratory population housed by Dr. X. Bellés’ group at the Institute of Evolutionary Biology (CSIC-UPF, Barcelona, Spain) was reared in plastic jars with aeration inside climatic chambers at the Institute for Integrative Systems Biology (University of Valencia—CSIC) at 25 °C, 60% humidity and photoperiod of 12 h light/12 h darkness. Insects were fed dog-food pellets (Teklad global 21% protein dog diet 2021C, Envigo, Madison, USA) and water ad libitum. Rifampicin (0.1 mg/mL) (Alfa Aesar, Kandel, Germany) was supplied in the water when needed.

### 2.2. Experimental Design

**Figure 1:**
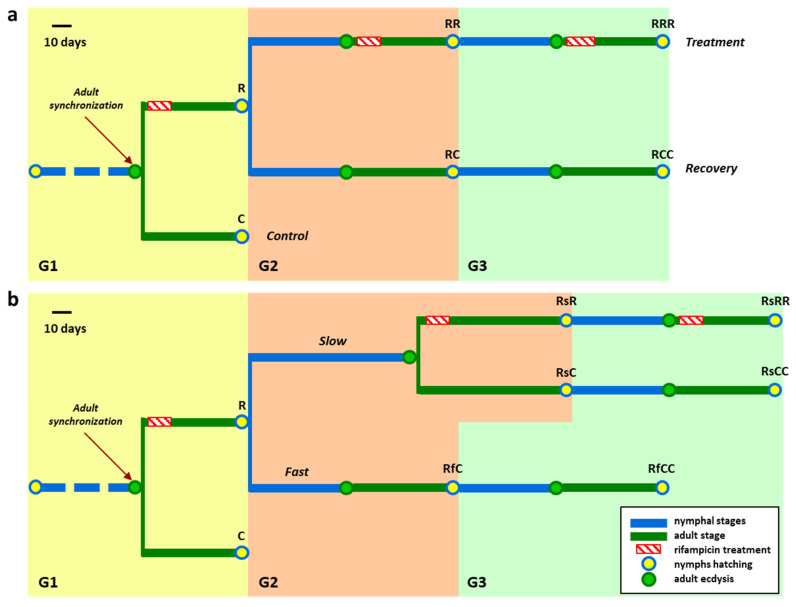
Experimental design to obtain *B. germanica* quasi-aposymbionts without affecting the gut microbiota. (**a**) Original design. The antibiotic rifampicin is supplied to a synchronized adult population of *B. germanica* in three successive generations. At G1, the following two populations are established: a control one without antibiotic (C) and another one treated with rifampicin for the first 12 days of the adult stage (R). At G2, within 48 h of hatching, nymphs of the antibiotic-treated population are used to start two new populations, with and without antibiotic treatment in the adult stage (RR and RC, respectively). At G3, the antibiotic is supplied again to the treated population only (RRR) in the adult stage, while the sister population remains untreated (RCC) to allow a complete recovery. (**b**) Modified design. Because the nymphs that hatched after the first rifampicin treatment present two developmental phenotypes (slow and fast), only those with a delay in their developmental time were used for the rifampicin treatment over three generations, and to analyse their recovery after ceasing the antibiotic treatment for two generations. Adult females were taken for dissection after the hatching of nymphs in G2 and G3.

Figure 1 presents a scheme of the experimental design and a summary of all samples analysed. The experiment is designed to span several generations to reduce the endosymbiont population progressively (Figure 1a). Between 0 and 48 h after ecdysis, adult cockroaches were collected to start a sex-balanced synchronized population. This first generation (G1) was distributed into the following two subpopulations: one was not treated with the antibiotic and served as a control (C), and the other (R) was treated with rifampicin 0.1 mg/mL for 12 days. At the second generation (G2), within 48 h of hatching, nymphs of the antibiotic-treated population were used to start two new subpopulations, which were antibiotic-treated (RR) or not (RC) at the adult stage. In the third generation (G3), the same treatment as the corresponding G2 was applied, leading to populations RRR and RCC.

During G2, some individuals developed normally, while others were delayed. For this reason, on day 20 the insects were separated based on this “developmental speed” in two groups, fast (Rf) and slow (Rs) (Figure 1b). When becoming adults, subpopulation Rf was maintained without treatment (RfC), while Rs was split into two halves, untreated (RsC) and antibiotic-treated (RsR). For the latter, an individualized system was designed in which each female reaching the adult stage was put in a container with a male, and the antibiotic was supplied for 12 days, after which all individuals with the same treatment were put together. The nymphs that hatched in G3 in each slow population were raised to adults, and were treated or not in the same conditions as their parents, giving rise to the populations RsRR, to continue with the treatment, and RsCC to check the recovery of the endosymbiont. A single untreated population was maintained for the fast population (RfCC).

### 2.3. Cockroach Dissections

At selected time points (Figure 1b), adult females were dissected after hatching their nymphs (i.e., after the oothecae were fully mature) to collect their hindguts and fat bodies. Only females were dissected to reduce statistical noise produced by sex bias. Cockroaches were anesthetized using a CO_2_ stream and dissected under stereomicroscope. The hindgut was isolated, opened and cleaned in Krebs–Ringer bicarbonate buffer (Sigma-Aldrich, USA) to remove faeces. The fat body was recovered in Krebs–Ringer buffer. Samples were frozen in liquid nitrogen and stored at −80 °C until used.

### 2.4. Fitness Parameters Determination

The following several fitness parameters were measured through the insect’s life-cycle: development (number of days from hatching to reach adult stage and to the ootheca hatching), reproductive capability (number of nymphs per ootheca), and viability (% nymphs’ survival in G2). Statistical differences were analysed with ANOVA and *t*-test, when appropriate.

### 2.5. DNA Extraction and Quantitative PCR

Fat body and hindgut total DNA were extracted following the protocols previously described [6]. Fat body DNA was resuspended in 50 µL of Milli-Q water, quantified with a Qubit 3.0 fluorimeter (Thermo Fisher Scientific, USA), and diluted up to 0.05 ng/µL. Endosymbiont population was analysed by qPCR on fat body samples using AriaMx real-time PCR system (Agilent Technologies, Germany). The genes *ureC* (accession number: NC_013454.1) and *actin5C* (accession number: AJ861721.1) were used as specific endosymbiont and host, respectively, using primers previously described (*ureC* quantification: UC1F, 5′-GTCCAGCAACTGGAACTATAGCCA-3′; UC1R, 5′-CCTCCTGCACCTGCTTCTATTTGT-3′. *actin5C* quantification: ActinF, 5′-CACATACAACTCCATTATGAAGTGCGA-3′; ActinR, 5′-TGTCGGCAATTCCGGTACATG-3′) [6], and the relative ratio *ureC*/*actin5C* was obtained. ANOVA and *t*-test were applied to analyse statistical differences.

### 2.6. DNA Sequencing and Bioinformatics Analyses

The V3–V4 region of the 16S rRNA gene was amplified by PCR from hindgut DNA with 16SrRNA Illumina sequencing standard primers (forward primer: 5′-CCTACGGGNGGCWGCAG-3′; reverse primer: 5′-GACTACHVGGGTATCTAATCC-3′), as described in [11], and sequenced with Illumina MiSeq at the Sequencing and Bioinformatic Service facilities of FISABIO. The obtained reads have been submitted to the European Nucleotide Archive (ENA), and the assigned study accession number is PRJEB47828 (ERS7664059-ERS7664084).

DNA reads were analysed using the QIIME2 platform [12], version qiime2-2019.7. After data demultiplexing, reads were denoised with DADA2 [13]. Taxonomic classification was performed using the SILVA 132 database [14]. Statistical analyses were performed using GNEISS and diversity plugins in QIIME2 and R packages.

Several biodiversity indexes have been calculated using plugins implemented in the QIIME2 pipeline [12]. Shannon index measures the bacterial distribution within each sample (alpha diversity). As for the analysis of beta diversity, Aitchison distance describes the dissimilarity between samples for compositional data [15,16], while weighted UniFrac takes into consideration the evolutionary relationship among the amplicon sequence variants (ASV) identified in each sample [17]. A principal coordinate analysis (PCoA) plot shows, in the axes, the parameters that best explain the distances between these bacterial communities.

## 3. Results and Discussion

In our previous work, we determined the putative functions of the gut microbiota, following a metagenomic approach [6,7]. However, the impact that those bacterial functions have on the host, and if they can somehow replace the endosymbiont’s essential functions, have not been proven. To empirically determine such effects, the use of hosts with a null or reduced load of *Blattabacterium* will be highly informative. Furthermore, they will also show how essential the endosymbiont is in the host’s development and fitness. Among the antibiotics previously tested in our group, to disturb the gut microbiota in *B. germanica*, only rifampicin can also affect *Blattabacterium*; its effect is observed in the following generation, as a reduction in the amount of endosymbiont in the offspring. It has been proposed that the antibiotic only affects the endosymbiont in the ovaries when it leaves the protection of the bacteriocytes to infect the oocytes, but not inside the bacteriocytes, except if high doses of antibiotic are used [18]. In the present experiment, rifampicin was supplied at half the concentration used in the previous work [5], and only for the first 12 days of the adult stage, with the aim of reaching the endosymbiont prior the oocytes’ invasion, while affecting the cockroaches’ fitness and gut microbiota as little as possible. It was designed this way because the antibiotic treatment also affects the gut community and could reduce insect viability, and the treatment must be maintained during several generations, in order to progressively reduce the endosymbiont load. In fact, the microbiota’s recovery is mainly expected by coprophagia once the antibiotic is removed [5,6,7], for which we expect there to be enough time because the females are dissected at least 20 days after the end of the treatment, i.e., the time necessary for the ootheca to hatch.

**Figure 2:**
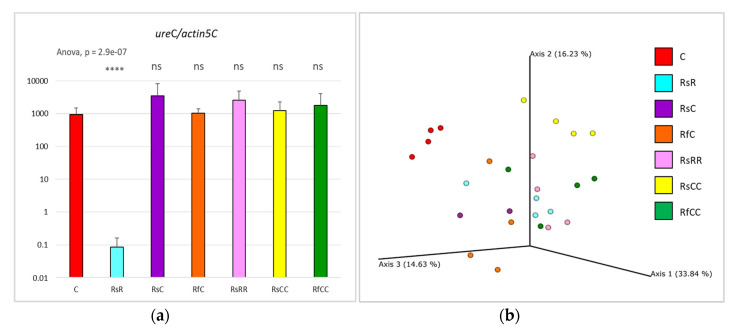
Effects of the rifampicin treatment on the two symbiotic systems of *B. germanica*. (**a**) Relative quantification of the endosymbiont in the fat body. Ratio *ureC*/*actin5C* in each sample of the analysed populations. ANOVA test comparing all the samples as a global *p*-value is shown in top left, and t-test values comparing each experimental sample to the control are shown above the bars (ns, *p* > 0.05; ****, *p* < = 0.0001). (**b**) PCoA analysis of the gut microbiota based on weighted UniFrac distances classified by sample type, indicating, on the axes, the percentages of variation explained by the principal coordinates.

The results of *Blattabacterium* quantification were striking (Figure 2a). The treatment for 12 days in G1 was able to reduce the endosymbiont population in some G2 individuals, suggesting that the antibiotic was affecting the endosymbiont during the infection of the ovaries, as expected [5]. However, this effect was not homogeneous, as we found two insect groups in G2, with different developmental times (fast and slow) (Figure 1b). Furthermore, the endosymbiont population was not equally reduced among the offspring of the treated cockroaches, and reduced amounts correlate with a slow development (less than 0.1 *ureC*/*actin* ratio in fat body DNA from the treated samples of the slow population, compared to 10^3^ in the control samples; Table 1). When the affected G2 was not treated again with rifampicin (RsC), the corresponding G3 population RsCC had normal *Blattabacterium* amounts. Surprisingly, we also observed that a G2 with a strong reduction in the endosymbiont population (RsR), treated again (RsRR), gave rise to a normal G3. This full recovery, even when the antibiotic was supplied in every generation, implies that the treatment window in G2 was not enough to have an effect on G3. Thus, it appears that the few individuals that reached the next generation were those that escaped the treatment. These results justify the need to keep a constant antibiotic stress, because in each ovary’s infection process, the endosymbiont population can grow again.

The differences in host development observed at G2 in our experiment can have several causes. First of all, despite starting with a synchronized population between 0 and 48 h after ecdysis, different adult individuals have a different time of development, following a normal distribution. Secondly, it must be taken into account that the G2 individuals born on 15 April did not come from a single ootheca, but from a collection of oothecae that hatched in the same 48 h. As the rifampicin is provided with water, it is possible that the treatment was not equally effective in different G1 females, and, therefore, their offspring would be differently affected regarding the *Blattabacterium* load. This wide variability in the samples had already been observed since the pioneering studies, in the second half of the 20th century, to obtain aposymbiotic cockroaches, where it was observed that in nymphs from the same ootheca, the amount of endosymbiont was not always equally affected by the antibiotic treatments [18].

**Figure 3:**
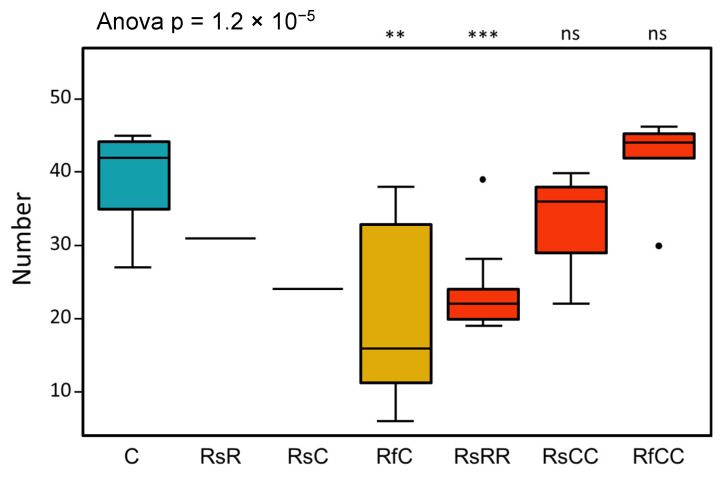
Number of nymphs per ootheca. ANOVA test comparing all the samples as a global *p*-value shown in the top left, t-test values comparing each experimental sample to the control shown above the bars (ns, *p* > 0.05; **, *p* <= 0.01; ***, *p* <= 0.001). T-test comparisons could not be performed in samples RsR and RsC due to the small number of replicates (under three), because a single ootheca hatched in these conditions. Blue box, control; yellow boxes, G2; red boxes, G3.

We checked if the reduction in *Blattabacterium* affects the nymphs’ viability and insects’ reproductive capability. During G1, we found no differences between the treated and control populations in any of the fitness parameters analysed (data not shown), as expected, because (as above stated) *Blattabacterium* is not affected during its intracellular stage in the fat body, but only during the infection of the ovaries and oocytes, and the treatment was applied in the adult stage. However, at G2, we observed a high mortality rate in nymphs from females treated with rifampicin (up to 90% mortality before becoming adults, while the control population had a mortality rate of 13.8%, SD 7.1). The reduction in the endosymbiont population was also accompanied by an extension of the host’s developmental time (up to 96 days to reach adult stage, instead of 50–70 days in normal conditions; Figure 1b, Table 1). Our hypothesis is that the endosymbiont reduction increases the developmental time, expanding the time window of the ovaries’ infection, which reduces the effectiveness of the treatment in the following generation with the same treatment regimen. The effects of supplying rifampicin for two or three consecutive generations clearly link the antibiotic treatment to a significant reduction in reproductive capacity, since the oothecae production and the number of nymphs per oothecae were affected in all the experimental groups treated, at least once, with the antibiotic (Figure 3). This decrease in reproductive fitness, however, cannot be attributed to the endosymbiont reduction, as it is detected in all the treated populations in G2 and G3, even with a normal amount of endosymbiont.

**Figure 4:**
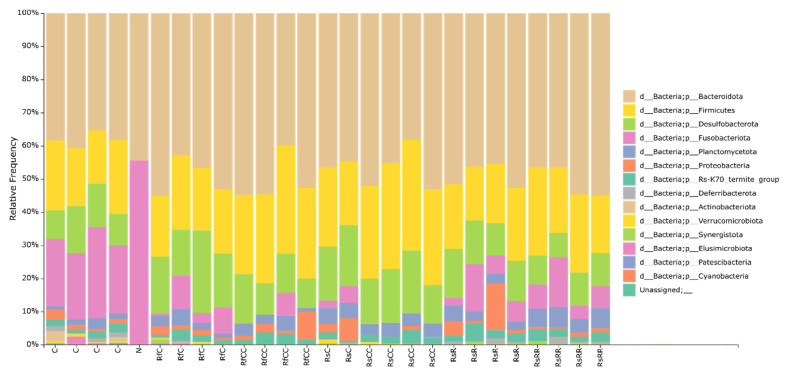
Bacterial composition of the *B. germanica* gut microbiota per sample at the phylum level. The most abundant taxa (>0.5%) are presented. The y-axis represents the proportion of sequencing reads belonging to each taxon. N, negative control (water).

We also studied the gut microbiota composition in all the samples in the experiment. A total of 2,599,001 reads were analysed, 78.03% of which passed the quality test. The taxonomic distribution was analysed at the phylum level (Figure 4). The most abundant phyla in all the samples correspond to Bacteroidota, Firmicutes, Desulfobacterota (previously included in the phylum Proteobacteria [19]), Proteobacteria, Fusobacteriota, and Planctomycetota, as previously reported [7]. A strong reduction in Fusobacteria was observed in most treated samples in G2 and G3, and it was the only bacterial genus that was not recovered when no antibiotic was added in the following generations (unnoticeable amounts in all the samples from population RsCC, and three out of four samples of population RfCC). This reduction does not correlate with the amount of *Blattabacterium,* which was normal in those samples. No significant effects on the host’s reproductive capability were noticed in these populations either. These findings are in consonance with previous results [5], where, in rifampicin-treated samples, a significant increase in Fusobacteria was found in G1, but was followed by a strong reduction during G2. Such changes could be explained by a dependence on the presence/absence of competitors during hindgut colonization [20].

Previous studies on *B. germanica* revealed a complex gut community with an interdependent structure, which can be highly affected by antibiotic treatments, but it is also very robust and recovers after antibiotic exposure [5,6,7]. All those studies used longer treatment periods, even during full generations. Thus, the lack of such strong effects observed here could be explained by the reduced treatment window and the long time given for recovery (at least 20 days after the treatment) before the sampling points. This indicates that this is a good strategy to reach our goal of affecting the endosymbiont, without a drastic effect in the gut microbiota.

**Figure 5:**
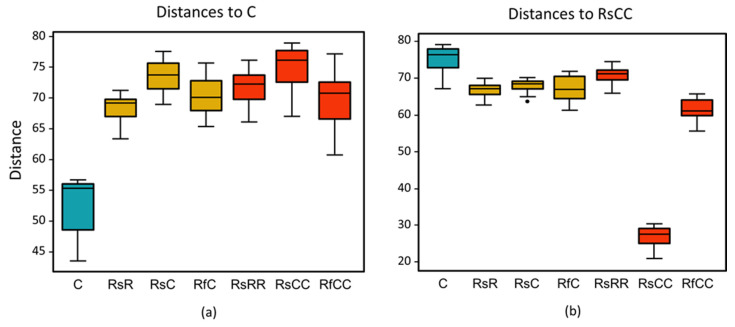
Aitchinson distances between all analysed gut samples and (**a**) the control, and (**b**) the RsCC population. Blue box, control; yellow boxes, G2; red boxes, G3.

**Figure 6:**
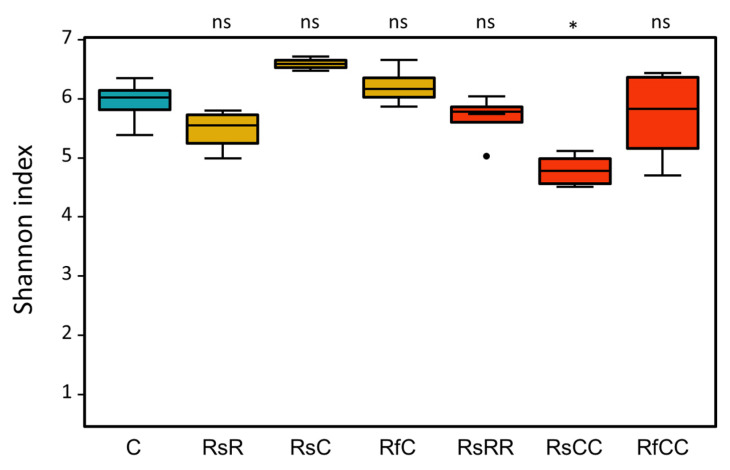
Alpha diversity (Shannon index) of the *B. germanica* gut microbiome of all experimental groups. The values of the t-test are shown above the bars (ns, *p* > 0.05; *, *p* <= 0.05). Blue box, control; yellow boxes, G2; red boxes, G3.

There were differences between the gut microbiota of all the experimental groups and the control population, as revealed by the Aitchison distance analysis (Figure 5), but they cannot be explained either by the antibiotic treatment or the amount of *Blattabacterium*. These results are reinforced by the analysis of the taxonomic distribution (weighted UniFrac PCoA; Figure 2b) and the alpha diversity (Shannon index; Figure 6), where the only significant differences were detected in the RsCC population.

A PERMANOVA test was applied to the Aitchison distance results by comparing each sample against all the others. The analysis revealed significant differences (p-value < 0.05) in all the comparisons with the control and RsCC populations (except for the RsC population, probably due to the small sample size). The corresponding Aitchison distances are presented in Figure 5. However, the weighted UniFrac PCoA (Figure 2b) shows only a slight separation of the control and RsCC population from the others. These results could indicate that, even though the re-established gut microbiota, after the rifampicin treatment in G1, is not exactly the same as in the control population, the taxonomical groups involved are quite similar and might be performing similar functions. Even though the case of the RsCC population is surprising, the changes in the microbiota composition are not associated with retardation in development, while the amounts of endosymbiont were normal in this population and, therefore, would not be useful to study a putative role of the gut microbiota to replace the endosymbiont. In addition, no interaction between the gut microbiota and the endosymbiont was detected, as there were no significant changes in G2 in the gut communities of the quasi-aposymbiotic populations, compared with those with a normal *Blattabacterium* load.

## 4. Conclusions

The selected treatment regimen (0.1 mg/mL of rifampicin for the first 12 days of the adult stage) has been successful in reducing the endosymbiont population of *B. germanica,* while affecting the gut microbiota as little as possible. However, its effect was not homogeneous among the cockroach population. Moreover, after one generation with reduced amounts of *Blattabacterium*, the offspring of the next generation recover completely, even though the antibiotic is supplied following the same schedule, probably because the extracellular stage of *Blattabacterium* has been delayed due to the developmental retard.

The negative effects on viability and reproductive fitness detected in rifampicin-treated populations are not related to changes in the gut microbiota, and cannot be attributed exclusively to the endosymbiont reduction, or to the rifampicin treatment itself. Moreover, apparently normal gut microbiota, recovered after cessation of the antibiotic treatment, are not able to replace the functions of *Blattabacterium*.

## Figures and Tables

**Table 1:** Female cockroaches of G2 and G3 analysed in this study. Columns indicate the dates (day/month/year) corresponding to relevant points in the developmental process (hatching, adult ecdysis, ootheca appearance, offspring hatching), number of days from hatching to reach adult stage and to next-generation hatching, reproductive capability (nymphs per ootheca), and endosymbiont population density (ratio *ureC/actin5C*).

Sample	Date Hatching	Date Adult Ecdysis	Date Ootheca	Date Offspring Hatching	Days to Adult	Days to Hatching	Nymphs/Ootheca	Ratio *ureC/actin5C*
RsR-1 ^a^	15/04/18	25/06/18	10/07/18	06/08/18	71	113	31	0.0932
RsR-2	15/04/18	26/06/18	_	_	72	_	_	0.0001
RsR-3	15/04/18	28/06/18	_	_	74	_	_	0.0723
RsR-4	15/04/18	03/07/18	_	_	79	_	_	0.1797
RsR-5	15/04/18	20/07/18	_	_	96	_	_	nd
RsC-1 ^b^	15/04/18	13/06/18	26/07/18	13/09/18	59	151	24	6868.99
RsC-2	15/04/18	09/07/18	_	_	85	_	_	16.802
RfC-1 ^c^	15/04/18	06/06/18	19/06/18	20/07/18	52	96	13	1470.02
RfC-2	15/04/18	09/06/18	21/06/18	26/07/18	55	102	16	1103.08
RfC-3	15/04/18	10/06/18	20/07/18	03/08/18	56	110	6	1015.49
RfC-4	15/04/18	14/06/18	20/07/18	10/08/18	60	117	33	514.67
RfC-5	15/04/18	18/06/18	20/07/18	16/08/18	64	123	33	nd
RsRR-1	06/08/18	10/10/18	22/10/18	27/11/18	65	113	39	2223.70
RsRR-2	06/08/18	22/10/18	16/11/18	11/12/18	77	127	19	1465.86
RsRR-3	06/08/18	29/10/18	16/11/18	14/12/18	84	130	28	735.42
RsRR-4	06/08/18	05/11/18	27/11/18	26/12/18	91	142	22	5872.96
RsRR-5	06/08/18	09/11/18	27/11/18	26/12/18	95	142	20	nd
RsCC-1	13/09/18	30/10/18	20/11/18	26/12/18	47	104	36	16.42
RsCC-2	13/09/18	03/11/18	27/11/18	26/12/18	51	104	30	2263.17
RsCC-3	13/09/18	06/11/18	26/12/18	03/01/19	54	112	30	2033.46
RsCC-4	13/09/18	20/11/18	26/12/18	03/01/19	68	112	22	520.99
RsCC-5	13/09/18	20/11/18	26/12/18	08/01/19	68	117	40	nd
RfCC-1	20/07/18	10/09/18	14/09/18	23/10/18	52	95	44	5106.13
RfCC-2	20/07/18	11/09/18	17/10/18	24/10/18	53	96	45	463.96
RfCC-3	20/07/18	12/09/18	17/10/18	26/10/18	54	98	46	1017.05
RfCC-4	20/07/18	13/09/18	17/10/18	26/10/18	55	98	44	504.08
RfCC-5	20/07/18	13/09/18	17/10/18	29/10/18	55	101	46	nd

^a^ All RsRR samples come from the RsR-1 offspring; ^b^ all RsCC samples come from the RsC-1 offspring; ^c^ all RfCC samples come from the RfC-1 offspring; a hyphen indicates that the corresponding female did not produce an oothecae; nd: non-determined. Each dark-coloured line on the second generation corresponds to the ootheca that served to start the next generation with the same colour.

## Data Availability

The obtained 16S rRNA reads have been submitted to the European Nucleotide Archive (ENA), and the assigned study accession number is PRJEB47828 (ERS7664059-ERS7664084).
